# Microbiome in the Apical Root Canal System of Teeth with Post-Treatment Apical Periodontitis

**DOI:** 10.1371/journal.pone.0162887

**Published:** 2016-09-30

**Authors:** José F. Siqueira, Henrique S. Antunes, Isabela N. Rôças, Caio T. C. C. Rachid, Flávio R. F. Alves

**Affiliations:** 1 Molecular Microbiology Laboratory, Department of Endodontics, Faculty of Dentistry, Estácio de Sá University, Rio de Janeiro, Rio de Janeiro, Brazil; 2 Department of Endodontics, Faculty of Dentistry, Grande Rio University, Duque de Caxias, Rio de Janeiro, Brazil; 3 Institute of Microbiology Prof. Paulo de Góes, Federal University of Rio de Janeiro, Rio de Janeiro, Brazil; Forsyth Institute, UNITED STATES

## Abstract

**Introduction:**

Bacteria present in the apical root canal system are directly involved with the pathogenesis of post-treatment apical periodontitis. This study used a next-generation sequencing approach to identify the bacterial taxa occurring in cryopulverized apical root samples from root canal-treated teeth with post-treatment disease.

**Methods:**

Apical root specimens obtained during periradicular surgery of ten adequately treated teeth with persistent apical periodontitis were cryogenically ground. DNA was extracted from the powder and the microbiome was characterized on the basis of the V4 hypervariable region of the 16S rRNA gene by using paired-end sequencing on Illumina MiSeq device.

**Results:**

All samples were positive for the presence of bacterial DNA. Bacterial taxa were mapped to 11 phyla and 103 genera composed by 538 distinct operational taxonomic units (OTUs) at 3% of dissimilarity. Over 85% of the sequences belonged to 4 phyla: Proteobacteria, Firmicutes, Fusobacteria and Actinobacteria. In general, these 4 phyla accounted for approximately 80% of the distinct OTUs found in the apical root samples. Proteobacteria was the most abundant phylum in 6/10 samples. Fourteen genera had representatives identified in all cases. Overall, the genera *Fusobacterium* and *Pseudomonas* were the most dominant. *Enterococcus* was found in 4 cases, always in relatively low abundance.

**Conclusions:**

This study showed a highly complex bacterial community in the apical root canal system of adequately treated teeth with persistent apical periodontitis. This suggests that this disease is characterized by multispecies bacterial communities and has a heterogeneous etiology, because the community composition largely varied from case to case.

## Introduction

Post-treatment apical periodontitis is a disease associated with root canal-treated teeth and is primarily caused by bacterial infection of the root canal system [1]. Bacteria enduring root canal treatment procedures are the main causative agents of this disease [2]. Culture-dependent and -independent studies have evaluated the bacterial communities associated with post-treatment apical periodontitis and identified some potential candidate pathogens [3–10]. In general, a lower bacterial diversity occurs in association with post-treatment apical periodontitis in comparison with the primary disease. *Enterococcus faecalis* has been the most frequently detected species in many studies [3–5, 7–8, 11–14], but other species have been encountered in what has been revealed to be a mixed infection [5–7, 9].

Four generation of studies have largely contributed to the knowledge of bacterial endodontic infections [15]. Recently, a 5th generation has arisen based on next-generation DNA sequencing (NGS) approaches, which provide a large number of sequence reads per run, resulting in very large sampling depth and coverage, and allowing for the detection of not only the most dominant bacterial community members, but also the low-abundance taxa [16–19]. Some studies have previously used NGS methods to assess the microbiome associated with post-treatment apical periodontitis. Studies using pyrosequencing (a NGS approach) reported a significantly higher bacterial diversity in symptomatic and asymptomatic root canal-treated teeth in comparison with the 4 previous generations of endodontic microbiology studies, with dominance of members from the phyla Firmicutes, Proteobacteria, Bacteroidetes and Actinobacteria [20–22]. A study used Illumina sequencing (another NGS technology) to analyze different types of endodontic infections and found polymicrobial communities in all, with dominance of taxa belonging to the phyla Firmicutes and Bacteroidetes [23].

The large majority of the previous studies dealing with the microbiota of treated root canals used the traditional paper point approach to obtain samples from the canal system. In clinical studies, paper point samples are taken from the entire extent of the main root canal, not permitting to infer in which segment of the canal the detected species are located. Because most bacteria causing post-treatment apical periodontitis occur in the apical root canal system [24–27], it is important to restrict identification to bacteria present in this specific region. Moreover, bacteria associated with post-treatment disease are often present in areas that are virtually inaccessible to instruments and irrigants, such as lateral canals, apical ramifications, isthmi and dentinal tubules [24–28], in which paper points are not expected to reach either. A different approach has been recommended to sidestep these limitations of the paper point approach [29]. By cryopulverizing the apical root fragment of extracted teeth or specimens obtained during periradicular surgery, one can include in the sample bacteria present selectively in the apical canal and in every location of the system. A study has used pulverization of the root apex from treated teeth to quantify the total bacterial load and the levels of candidate endodontic pathogens in this critical region [30].

The present study was intended to evaluate the apical root canal microbiome of teeth with post-treatment apical periodontitis. Only teeth with the previous treatment regarded as adequate on the basis of radiographs and cone beam computed tomography (CBCT) were included. Root specimens were obtained by root-end resection during periradicular surgery, were pulverized in a cryogenic mill, and then subjected to a NGS technology (Illumina sequencing).

## Material and Methods

### Case description

Samples were obtained from 10 patients (7 female and 3 male; aged 38 to 62 years; mean age 50 years) who had been referred to a private practice from one of the authors (H.S.A.) in need of periradicular surgery due to post-treatment apical periodontitis. Each patient contributed one root canal-treated tooth, which was asymptomatic and presented with an apical periodontitis lesion as determined radiographically and confirmed by CBCT (requested for surgery planning). The initial root canal treatments/retreatments were performed by dentists other than those involved in this study; clinical information and radiographs taken at the time of the previous treatments were available for surgical indication. Other inclusion criteria were: previous root canal treatment or retreatment performed at least more than 1 year before; the lesion remained the same size or increased as compared with radiographs taken immediately after the initial treatment/retreatment; teeth with satisfactory coronal restorations and no direct exposure of the root canal filling material to the oral cavity; teeth with no periodontal disease; and teeth with adequate root canal treatments on the basis of the apical terminus (from 0 to 1 mm short of the apex), homogeneous fillings (no voids) and tapered canal shape, as determined both radiographically and by CBCT analysis. The study protocol was approved by the Ethics Committee of the Estácio de Sá University and written informed consent was obtained from all individuals.

### Sample taking and cryopulverization

Root apex specimens were obtained during periradicular surgery. Before surgery, the oral cavity was rinsed with 0.12% chlorhexidine for 2 minutes and the area to be operated was gently scrubbed with the same solution. An intrasulcular incision was used to reflect a full-thickness mucoperiosteal flap. Care was taken to avoid saliva contamination of the surgical site during flap elevation and handling. After curettage, the lesion specimen was placed in 10% buffered formalin solution for histopathologic examination (7 cases were diagnosed as granuloma and 3 as cyst). Root-end resection was carried out using a sterile Zekrya FG 28mm bur (Maillefer, Ballaigues, Switzerland) under copious sterile saline irrigation. A 3- to 5-mm long fragment of the root apex was obtained and after being rinsed with sterile saline, it was freed of attached soft tissue remnants by using a #15 sterile surgical scalpel. The apical root fragment was placed in a sterile flask and immediately frozen at -20°C. Surgery was completed by root-end preparation with ultrasonic tips and root-end filling with a bioceramic material. An operating microscope was used throughout the surgical procedures.

Later, the root fragments were thawed and had their outer surfaces cleaned and disinfected with 3% hydrogen peroxide and 2.5% sodium hypochlorite (NaOCl), respectively. Ten percent sodium thiosulfate was used to inactivate NaOCl and the external root surfaces were sampled by using a #80 sterile paper point dampened with TE buffer (10 mM Tris-HCl, 1 mM EDTA, pH 7.6). This sample served as sterility control and was assessed by single polymerase chain reaction (PCR) assay using universal 16S rRNA-gene primers [30]. Cleaning, disinfecting and control sample taking procedures were all conducted under an operating microscope. The root apexes were finally crushed in a 6750 freezer mill (Spex, Metuchen, NJ, USA), at the liquid nitrogen temperature, as reported elsewhere [29–31]. Apical root powder samples were stored frozen at -20°C. Later, the powder was suspended in TE buffer and DNA was extracted by using the QIAamp DNA Mini Kit (Qiagen, Valencia, CA, USA).

### 16S rRNA gene sequencing

PCR primers 515/806 based on the 16S rRNA gene V4 variable region [32], with the forward primer barcoded, were used in a 30-cycle PCR assay using the HotStarTaq Plus Master Mix Kit (Qiagen) under the following cycling parameters: 94°C/3 min, 28 cycles of 94°C/30 s, 53°C/40 s and 72°C/1 min, and final elongation at 72°C/5 min. Next, PCR products were run in a 2% agarose gel for evaluation of the presence of the predicted bands and their relative intensity. Samples were pooled together in equal proportions on the basis of molecular weight and DNA concentration. Calibrated Ampure XP beads were used to purify the PCR products, which were then used to prepare a DNA library according to the Illumina TruSeq DNA library preparation protocol. Paired-end DNA sequencing was carried out at the Mr DNA facility (www.mrdnalab.com, Shallowater, TX, USA) on an Illumina MiSeq device (Illumina Inc, San Diego, CA, USA). After quality q25 trimming of the ends with Mr DNA pipeline, the sequences from each end were joined.

Mothur v.1.36.1 [33] was used to process the resulting files. Primer and barcode sequences were cleaned from the sequences. The exclusion criteria included sequences with average quality lower than 30 (window size of 50), shorter than 200 bases long, with ambiguities, and with more than 1 nucleotide mismatch to the primer. Mothur with the SILVA reference database [34] was used used to align the sequences. Chimeras were detected with Uchime [35] and then removed. Sequences were taxonomically classified using the Naïve Bayesian supervised classification method, with 80% of confidence threshold. The Human Oral Microbiome Database (HOMD v13.2) was used as reference for identification. Sequences that were not classified into the Bacteria root were disregarded. Accordingly, high-quality sequences ranging from 252 to 254 bp were available. Random subsampling was done to normalize samples to the same sequence number (i.e., 7,558). A distance matrix was constructed and the sequences clustered into operational taxonomic units (OTUs) at 3% dissimilarity cut-off. Sequences obtained from the 10 clinical samples are available at the NCBI Sequence Read Archive under the accession number SRP075560.

All singletons were removed to increase the confidence in the generated clusters. Species-richness estimators and the Shannon diversity index were calculated using the remaining clusters. Sequences and OTUs were taxonomically assigned as above and the relative abundance of each taxon in the samples was determined.

## Results

**Fig 1:**
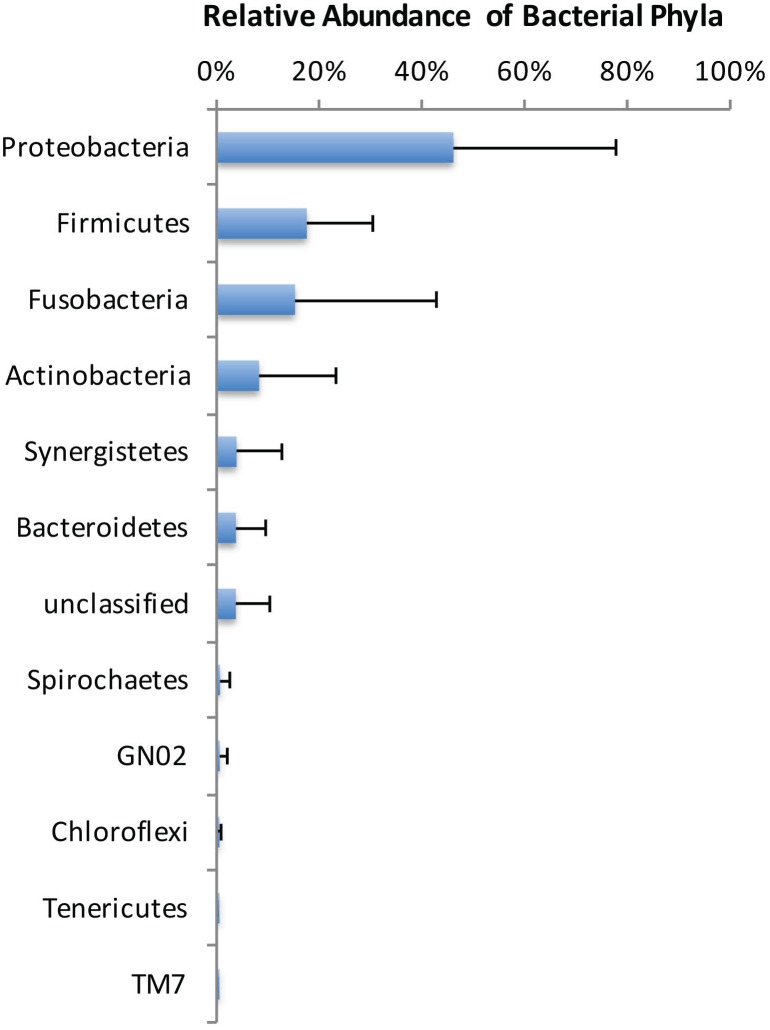
Average relative abundance of bacterial phyla composition in root apex samples from teeth with post-treatment apical periodontitis.

**Fig 2:**
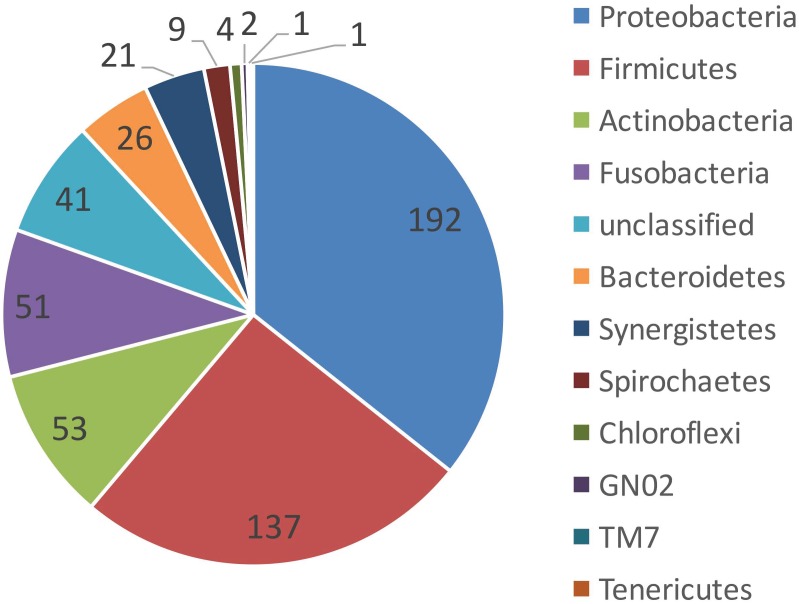
Taxonomic assignment (phylum level) of the operational taxonomic units found in root apex samples from teeth with post-treatment apical periodontitis.

**Fig 3:**
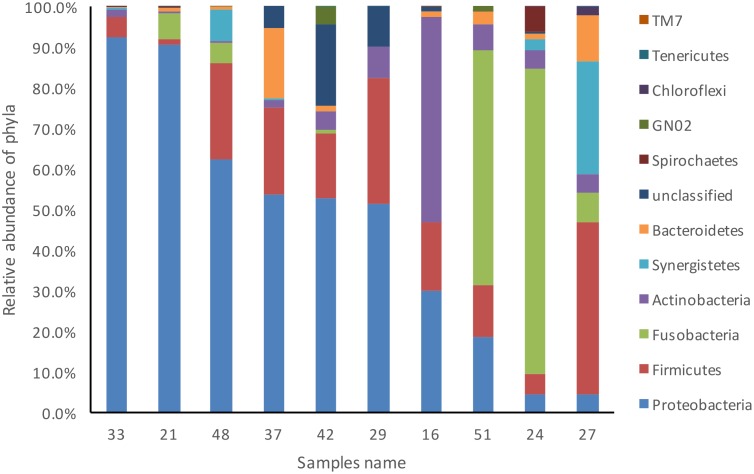
Relative abundance of bacterial phyla composition in each one of the 10 root apex samples from teeth with post-treatment apical periodontitis.

All sterility controls from the outer root surfaces yielded negative results. All 10 cryopulverized apical root samples were positive for the presence of bacterial DNA. Paired-end sequencing on Illumina MiSeq revealed a total number of 285,119 partial 16S rRNA gene sequences. After subsampling for normalization, 75,580 sequences that passed the quality control were used for analyses (7,558 sequences per sample). Bacterial taxa were mapped to 11 phyla and 103 genera composed by 538 distinct OTUs with >3% of dissimilarity. Over 85% of the sequences belonged to 4 phyla: Proteobacteria (46%), Firmicutes (18%), Fusobacteria (15%) and Actinobacteria (8%) (Fig 1). In general, these phyla were also the most represented, accounting for approximately 80% of the distinct OTUs found in the apical root samples (Fig 2). Forty-one OTUs could not be assigned to any bacterial phylum, but these collectively accounted to less than 4% of the community. Proteobacteria was the most abundant phylum in 6 of the 10 samples, Fusobacteria dominated 2 samples, and Firmicutes and Actinobacteria were the most dominant in 1 sample each (Fig 3).

**Fig 4:**
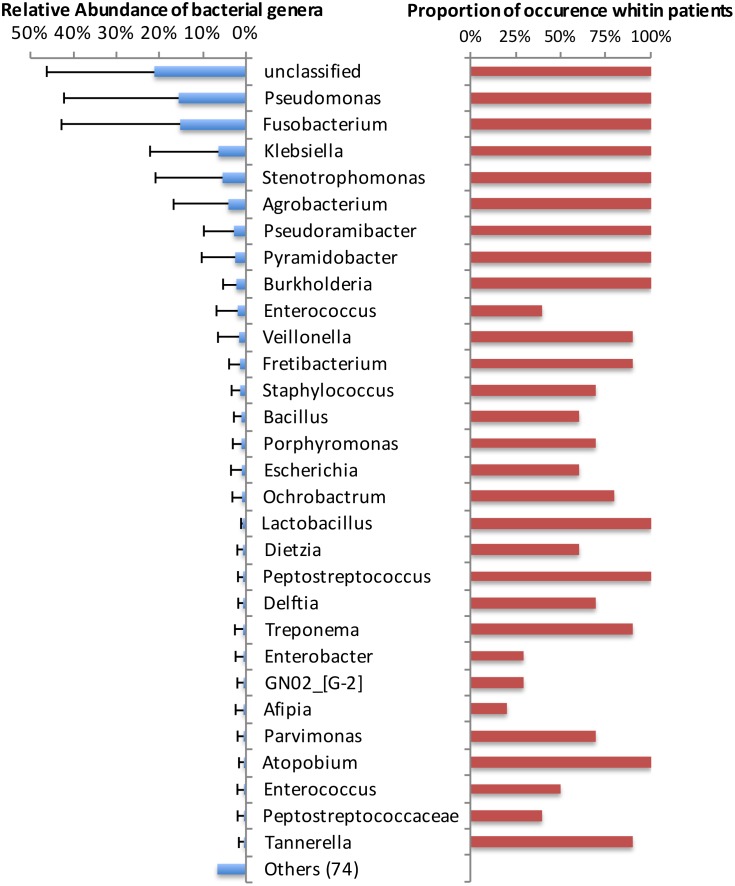
Average relative abundance of the top most abundant bacterial genera (left bars), and proportion of occurrence among the root apex samples from teeth with post-treatment apical periodontitis (right bars).

**Fig 5:**
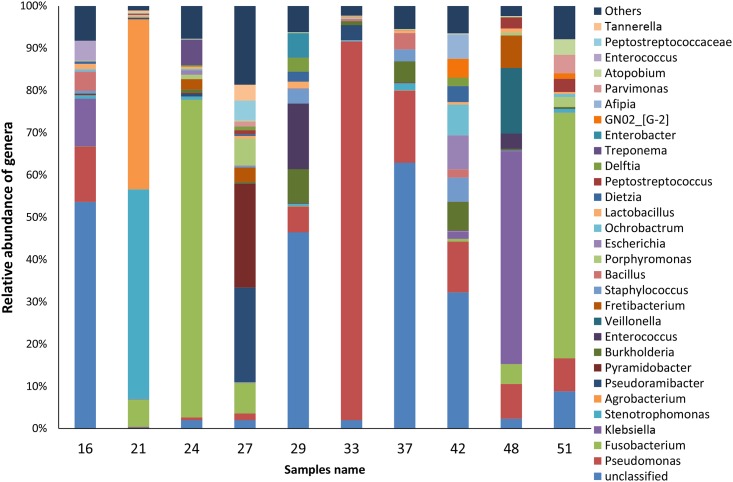
Relative abundance of the top most abundant bacterial genera in each one of the 10 root apex samples from teeth with post-treatment apical periodontitis.

Fourteen genera had representatives identified in all cases (Fig 4). Two of them (*Olsenella* and *Pseudoxanthomonas*) are shown as "others" in Fig 4, because they occurred only in very low levels (<0.8%). Overall, the genera *Fusobacterium* and *Pseudomonas* were the most dominant, each accounting for 15% of the sequences (Fig 4). In terms of relative abundance per case, the most dominant genera were "unclassified" (4 samples), *Fusobacterium* (2 samples), *Pseudomonas* (1 sample), *Pyramidobacter* (1 sample), *Stenotrophomonas* (1 sample), and *Klebsiella* (1 sample) (Fig 5). *Enterococcus* was found in 4 cases, always in relatively low abundance (mean, 1.9%; range, 0.01 to 15.5%). About 21% of the sequences could not be classified at the genus level.

The mean number of distinct OTUs at 3% dissimilarity present per apical root sample was 116 (range, 86 to 146). Table 1 depicts data from diversity calculations. Chao and ACE nonparametric measures of richness revealed that there is a predicted mean of 210 and 343 distinct OTUs per apical root sample, respectively. The Shannon index, which takes into account the species richness and evenness, was calculated per sample and is also shown in Table 1.

**Table 1:** Richness and diversity indexes of bacterial community in the apical root canal system.

Sample	OTUs	Chao	Ace	Shannon Index
16	137	228	313	2.22
21	146	401	785	1.23
24	107	163	204	1.40
27	139	266	516	2.72
29	103	197	348	2.85
33	100	152	244	0.75
37	86	129	170	2.68
42	110	142	194	3.30
48	134	251	391	1.95
51	97	166	264	1.95

## Discussion

This study evaluated the microbiome occurring at the apical root canal system of teeth with post-treatment apical periodontitis and showed mixed bacterial communities with high bacterial diversity. This information has an impact on the understanding of the bacterial taxa involved with the persistent endodontic disease process and opens avenues for future research on the establishment of preventive (during initial treatment) or therapeutic (during retreatment or surgery) protocols.

Representatives of 11 bacterial phyla were identified in the apical root canal system. The most diverse and abundant phyla were Proteobacteria, Firmicutes, Fusobacteria and Actinobacteria. Members of the former were found to dominate 60% of the cases. Previous NGS studies of persistent/secondary infections reported the occurrence of up to 28 phyla [20–22]. A pyrosequencing study found Firmicutes, Proteobacteria, and Actinobacteria as the most dominant phyla [20], while two other studies reported that Bacteroidetes was the most abundant [21–22]. Enrichment of Proteobacteria in persistent/secondary infections was shown by Tzanetakis et al. [22]. Vengerfeldt et al. [23] analyzed different types of endodontic infections and found Firmicutes and Bacteroidetes to dominate. Although our findings are in general similar to the previous ones, we did not find Bacteroidetes as one of the most dominant phyla. This and other differences may be related to some aspects, including the distinction between adequate and inadequate previous treatments (which was not done in most previous NGS studies), the exclusive analysis of the apical canal (in contrast to paper point sampling used in the previous studies), geographical differences, and distinct analytical approaches, including the reference database.

The present study identified 538 different OTUs with >3% dissimilarity, belonging to 103 genera, 14 of which were represented in all samples. Overall, the most dominant were *Fusobacterium* and *Pseudomonas*. On a case-to-case basis, *Fusobacterium* dominated the community in 2 teeth, while *Pseudomonas*, *Pyramidobacter*, *Stenotrophomonas*, and *Klebsiella* dominated 1 tooth each. The other 4 teeth were dominated by bacteria that could not be identified to the genus level. Previous studies also found a high number of genera: Hong et al. [21] identified 133, Tzanetakis et al. [22] 347, and Anderson et al. [20] 525. These big differences between studies are likely to be related to the number of samples examined, the depth of sequencing, the analytical methods for identification, quality of previous fillings, and the selected region sampled (whole main canal in the paper point approach versus apical canal in the cryopulverization approach). *Fusobacterium* was also one of the most abundant genus in teeth with persistent/secondary infections in a previous study [22].

*E*. *faecalis* has been commonly found in post-treatment disease, with prevalence reaching up to 90% of the cases [5, 12]. However, some recent studies have shown a much lower frequency for this species [6, 9, 36]. The present study using an open-ended analysis identified members of the *Enterococcus* genus in 4 cases and in low abundance. The reasons why enterococci have been recently detected in lower prevalence and numbers remain uncertain, but one related to this study may have been the exclusive analysis of the apical canal. The possibility exists that the ecological conditions in this region is not favorable to the establishment of enterococci. Using cryopulverization of root-end resection specimens evaluated by quantitative real-time PCR, Antunes et al. [30] detected *E*. *faecalis* in only 14% of the samples. The findings of enterococci in only a few root apex specimens and in low abundance question the role of these bacteria as the main pathogen of post-treatment apical periodontitis.

The number of bacterial species in adequately treated teeth with post-treatment disease has been shown to be 1 to 2 by culture [3–4], and 1 to 5 by early-generation molecular methods [3–6, 37]. Inadequately treated teeth have been shown to harbor more species per case; i.e., more than 3 species in culture [4] and 10 to 30 in molecular studies [38–41]. NGS studies have found a substantially larger bacterial richness per tooth. Using Illumina sequencing in this study in an analysis restricted to the apical canal, we observed 116 distinct OTUs with >3% of dissimilarity per apical root sample, ranging from 86 to 146. Previous NGS studies found even larger numbers, i.e., 122 [21] and 162 [22] OTUs when evaluating the entire main canal. NGS have the great advantage over other methods of detecting even low-abundance or rare species [18] and this may be one of the reasons for the larger numbers of detected OTUs in comparison with other methods. However, if differences are also a result of analytical artifacts during PCR, filtering sequences or identification, or due to DNA free in the root canal environment, remains to be elucidated. How long DNA from dead cells can remain detectable in an environment with other living bacterial cells in a biofilm community has been focus of intense debate [42–43], and requires further elucidation.

Some genera not typically found in oral and endodontic infections, such as *Pseudomonas*, *Klebsiella* and *Stenotrophomonas*, were detected in this study. This may suggest occurrence of secondary infections in some cases, probably as a consequence of a breach in the aseptic chain during treatment. Whereas all surgeries were performed by the same operator in a controlled clinical environment, all the original root canal treatments/retreatments were done by other dentists under unknown clinical conditions. The quality of the root canal fillings usually serves as a surrogate measure for the quality of the overall treatment [44], but it cannot ensure that asepsis was followed during treatment. Risks for secondary infection with unusual bacteria are increased when: rubber dam isolation is inadequate or not even used; tap water is used for irrigation; irrigants such as saline are contaminated; instruments are touched with the fingers; the canal was left open to the oral cavity between appointments; and treatment was performed in multiple visits [45–46].

There are several important aspects of the present study design that makes it unique for its purposes. First, the study evaluated the microbiome present exclusively in the apical root canal system. Bacteria located in this region are in a strategic position to inflict damage to the host to induce and maintain periradicular inflammation. Indeed, morphologic studies revealed that the large majority of teeth with post-treatment apical periodontitis had bacterial infection in the apical canal system [24–27]. Previous studies using paper point sample taking could not discern in which part of the canal the identified bacteria were present. The analysis selectively restricted to the apical canal system was only made possible by making use of the cryopulverization approach. Only two previous studies have used this approach associated with NGS technologies in endodontic microbiology research, but both investigated the microbiome of primary infections (untreated teeth)[47–48].

Second, the cryopulverization approach also permits for analysis of the microbiome present not only in the main root canal, but also in other irregularities of the root canal system. The paper point approach is limited by its ability to sample only the bacterial cells present in the main canal and its immediate vicinity. Numerous studies have demonstrated that persistent bacteria associated with post-treatment disease are preferentially located in such irregularities [24–27].

Third, all teeth included in this study had their previous root canal treatments/retreatments rated as adequate on the basis of radiographs and CBCT scans used to evaluate the apical extent, homogeneity and taper of the fillings. In spite of the apparently adequate quality of the previous treatment/retreatment, lesions were classified as persistent and requiring further surgical intervention on the basis that they increased or remained the same size since the previous endodontic intervention [1]. Studies have observed different community compositions with higher diversity for infections associated with inadequately treated teeth when compared with treatment following acceptable standards [4–5]. By detecting and identifying bacteria present in adequately treated teeth, one can have an insight into the bacterial taxa that may have persisted after the previous treatment and may be the primary focus of attention in future research efforts.

Fourth, this study comprised an open-ended analysis using a NGS approach. This permits to detect unexpected taxa and reveal the bacterial diversity in the persistent/secondary endodontic infection process. NGS methods that permit massive DNA sequencing with a high throughput have been recently applied to the study of endodontic infections [16]. NGS technologies provide deeper coverage of bacterial identification when compared to previous approaches, such as the conventional Sanger sequencing technique [49].

The several advantages of this study design were addressed, but it is not certainly free of limitations. An important one is shared with many others also using NGS technologies and relates to the short 16S rRNA gene sequences that were used for bacterial classification. Therefore, the lower taxonomic level that bacteria were identified and reported herein was genus. The cryopulverization approach has also its limitation as it can only be used to process extracted teeth or teeth subject to root-end resection during periradicular surgery. There is also a risk for contamination of the outer root surface when obtaining or handling the specimen, so careful disinfection of the surface and sterility controls need to be taken. A side effect of the external disinfection is that bacteria present in biofilms outside the root canal system and adhered to the outer root surface will be killed and not detected. These extracanal bacteria may be the cause of the post-treatment disease in some teeth [50–52].

In conclusion, this study showed a highly complex bacterial community in the apical root canal system of adequately treated teeth with persistent apical periodontitis. This suggests that this disease is characterized by multispecies bacterial communities and has a heterogeneous etiology, since the community largely varied from case to case.
